# Relationship between Breastfeeding and Malocclusion: A Systematic Review of the Literature

**DOI:** 10.3390/nu12123688

**Published:** 2020-11-30

**Authors:** Andrea Abate, Davide Cavagnetto, Andrea Fama, Cinzia Maspero, Giampietro Farronato

**Affiliations:** 1 Department of Biomedical, Surgical and Dental Sciences, School of Dentistry, University of Milan, 20100 Milan, Italy; andreabate93@gmail.com (A.A.); davide.cavagnetto@gmail.com (D.C.); andreas.fama@hotmail.it (A.F.); giampietro.farronato@unimi.it (G.F.); 2 Fondazione IRCCS Cà Granda, Ospedale Maggiore Policlinico, 20100 Milan, Italy

**Keywords:** malocclusion, cross-bite, breastfeeding, orthodontics

## Abstract

Background: The purpose of this systematic review was to analyze the available literature about the influence of breastfeeding in primary and mixed dentition on different types of malocclusions. Methods: Preferred Reporting Items for Systematic Reviews and Meta-Analyses Protocols (PRISMA-P) guidelines were used to perform the present review. The following electronic databases were searched: Pubmed, Evidence-Based Medicine Reviews (EBMR), Embase, Cochrane Library, Medline, Web of Science and Ovid. Results: A primary research found a total of 279 articles. Two more papers were also considered from the gray literature. Two hundred sixty-three articles were excluded as they were deemed irrelevant on the basis of: duplicates, title, abstract, methods and/or irrelevant contents. Eighteen papers were selected and included in the qualitative analysis. Conclusions: breastfeeding is a positive factor that seems to reduce the incidence of posterior crossbite, skeletal class II and distoclusion in primary and mixed dentition. A sort of positive relationship between months of breastfeeding and risk reduction seems to exist. More longitudinal research is needed to avoid bias in the results, with data collected prospectively on the months of exclusive breastfeeding, by means of specific questionnaires and successive clinical evaluation of the occlusal condition at the primary dentition, mixed dentition and permanent dentition stages.

## 1. Introduction

The stomatognathic system is composed of static and dynamic structures and its harmonious functioning is based on the balanced relationship between them. The functions that include the stomatognathic apparatus (sucking, respiration, speech, chewing, swallowing) are believed to be the factors that most influence the model of maxillofacial development and the position of the teeth in the child’s arch [1,2,3,4]. The genesis of a malocclusion is usually linked to an impairment of some kind to eugnathic growth that involves to various extents the mandible, the maxilla [5], and the functional matrix (tongue and facial muscles). Exclusive breastfeeding between 0 and 6 months is recommended by the World health Organization (WHO) as a public health policy because it reduces the risk of aerodigestive infections [6,7,8]. Moreover, breastfeeding is one of the cornerstones of a correct maxillofacial growth because it promotes proper lip seal, mandibular function and tongue correct position against the palate [9]. Breastfeeding forces the child to actively squeeze milk out of the mother’s breast through a synergic action of both tongue and facial muscles, whereas bottle-feeding requires less effort to drain the milk, thus under stimulating the functional matrix [10,11,12,13].

The suction reflex is the first coordinated muscular activity performed by the newborn.

In the literature there are two forms of suction described: nutritive (breastfeeding and bottle-feeding) and non-nutritive suction [14,15,16]. The first one provides the child with the essential nutrients for their optimal development and growth; in particular breastfeeding is considered the best source of nutrition that a mother can offer her newborn child [17,18,19,20,21,22,23]. Breastfeeding is a nutritive sucking habit that lowers the incidence of malocclusion in the primary dentition [19,24,25,26,27]. Some studies found that prolonged breastfeeding seems to lower the incidence of malocclusion [3], others did not find this association [19,28,29,30,31,32]. Furthermore, minimum duration of breastfeeding to effectively protect against malocclusion is currently under debate. In fact, some authors suggest that 6 months [33,34] are enough while others recommend up to 12 months of breastfeeding. Several studies have investigated the connection between occlusion and breastfeeding and they often came to different conclusions. Some studies [15,29] found no evidence of a connection between breastfeeding duration and malocclusion while others were able to define a precise relationship between reduced or no breastfeeding and the development of different kinds of malocclusion, i.e., skeletal class II [35], open bite [36] or posterior crossbite [37,38,39].

The aim of the present paper is to systematically review the present evidence on the relationship between breastfeeding and the development of malocclusion traits during childhood in primary and mixed dentition, taking into consideration whether the duration of breastfeeding is a relevant factor for the onset of the same.

## 2. Materials and Methods

The protocol was registered in the Prospective International Registration of Systematic Reviews (PROSPERO) database under the reference number CRD42019137471.The research refers to the Preferred Reporting Items for Systematic Reviews and Meta-Analyses (PRISMA-P) 2015 [40].

### 2.1. Population, Intervention, Comparison, Outcome (PICO)

The PICO model was designed as follows: Population—subjects with primary or mixed dentition; Exposure—breastfeeding duration, presence of non-nutritive sucking habits, use of pacifier; Comparison—absence of breastfeeding, duration of breastfeeding less than 6 months; Outcome—the prevalence of any malocclusion diagnosed through patients’ objective examination, questionnaires or stone casts examination either in the transversal dimension (i.e., maxillary crowding or maxillary hypoplasia), in the vertical dimension(i.e., augmented or reduced anterior and/or posterior vertical dimension, skeletal and dental open or deep bite) and in the sagittal dimension that is skeletal and/or dental discrepancies between the mandible and maxilla (i.e., class 2 division 1 and division 2 according to Angle, primary canine distocclusion according to Foster and Hamilton, skeletal class 2 according to Steiner, augmented or reduced overjet, underdevelopment of the mandible)

### 2.2. Search Strategy

A rigorous electronic research was employed through the following electronic databases: Medline, Pubmed, Embase, Cochrane Library, Evidence-Based Medicine Reviews (EBMR), Web of Science and Ovid.

The following search strategy was conducted: (child* OR infant OR infant, newborn OR baby) AND (“breast feeding” (MeSH Terms) OR (“breast” (All Fields) AND “feeding” (All Fields)) OR “breast feeding” (All Fields) OR “breastfeeding” (All Fields)) AND (“malocclusion” (MeSH Terms) OR “malocclusion” (All Fields))) AND “humans” (MeSH Terms)). Hand searching in the references of included papers was performed to eventually retrieve any study that was not identified during the primary search. Two independent reviewers assessed the titles and abstracts of all the articles selected.

The EndNote software reference manager (Version X7 × 9.21, Thomson Reuters, released September 2014, Toronto, ON, Canada) was adopted to archive and analyze retrieved references studies.

The Kappa score [41] was chosen to evaluate agreement between the reviewers on the eligibility of retrieved results after reading the title and abstract. If the title and abstract was deemed insufficient to come to a decision, the full article was read to reach the final decision. A third researcher’s opinion was requested in case of disagreement between the two reviewers.

### 2.3. Inclusion and Exclusion Criteria

Articles with proper description of the diagnostic method used, information about types of nutrition in neonates, use of nonnutritive sucking habits (i.e., use of pacifier, thumb sucking, tongue thrust, and finger sucking), duration of breastfeeding and diagnosis of malocclusion in the primary/mixed dentition were considered only. Moreover, no time limits in the search strategy were considered. The following article types were included: randomized controlled trials (RCTs), case-control studies and cohort studies. Papers that evaluated the present evidence on the connection between breastfeeding and malocclusion during childhood were included. Studies in a language other than English, animal studies, reviews, comments, conference abstracts, personal opinions, book chapters, letters to the editor, and studies with insufficient information about how the data were collected were excluded.

### 2.4. Data Extraction

The following data were gathered from each of the selected papers: journal and publication date, sample size, gender, study design, malocclusion diagnosis, information on breastfeeding and on the odds ratio (OR) between breastfeeding and malocclusion, instrument and time Interval of feeding habit evaluation and therapeutic outcomes.

### 2.5. Quality Appraisal

Newcastle-Ottawa Quality Assessment Scale (NOS) [42] for cohort and case-control studies was used for qualitative analysis of selected studies. The aforementioned quality appraisal tool is made of 8 points. Each paper can be rated with only one star for each point, besides Comparability that can be rated up to two stars. The maximum score is, therefore, nine stars. Quality assessment was performed independently by the same investigators that performed the literature search and study selection. In case of dispute, the third reviewer’s opinion was asked.

### 2.6. Limitations of the Review

Because of the heterogeneity of study protocols, meta-analysis of the retrieved data was not possible. Therefore, only a qualitative analysis of retrieved studies was possible.

## 3. Results

**Figure 1:**
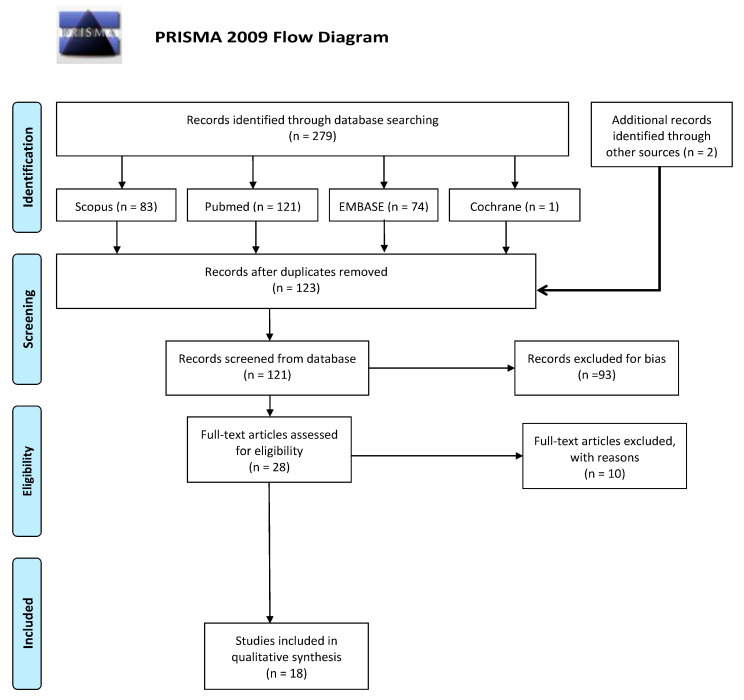
Preferred Reporting Items for Systematic Reviews and Meta-Analyses Protocols (PRISMA) flow chart.

The protocol registered with PROSPERO stated that this study would consider only papers published after the year 2000. Initially 279 articles were found in total. Two more papers from the gray literature were also included. The primary search retrieved 123 papers net of duplicates. Two papers were deemed excluded after reading the abstract, the title and the study design. Ninety three articles were excluded for mixed reasons (generation of random sequences; allocation concealment; blinding of participants and staff, blinding of the evaluation of results; incomplete outcome data; selective reporting; other prejudices.). Twenty eight papers were read in extenso, ten of them were excluded for lack of relevance. Eighteen articles were selected for qualitative analysis. Summary of clinical studies meeting inclusion criteria is shown in Table 1. The PRISMA flow chart (Figure 1) illustrates the search methodology and results.

### 3.1. Description of the Included Studies

Of the 18 papers selected, thirteen were cross-sectional, two cohort studies, two observational retrospective, one was a prospective study. The overall study sample was 11.827 children with a range between 80 and 2060 with an average age range of 3–5 years.

As regards to transverse skeletal malocclusion, 12 studies assessed the relationship between breastfeeding or bottle-feeding and maxillary hypoplasia (posterior crossbite). All of the studies, apart from Limeira et al. [37] and Sanchez Molins et al. [48] that assess the aforementioned association in mixed dentition, focused their attention on the primary dentition [14,15,18,38,39,44,50].

As regards to vertical discrepancy, 12 studies [3,10,14,15,18,34,36,38,39,44,45,48] investigated the relationship between breastfeeding and abnormal anterior overbite/openbite in deciduous teeth. Finally regarding sagittal discrepancy, seven studies [2,15,18,36,38,39,48] evaluated the relationship between breastfeeding and anterior crossbite in deciduous teeth.

Thomaz et al. evaluated the relationship between breastfeeding and prevalence of class II in mixed dentition, Caramez da Silva et al. in deciduous teeth. The relationship between the development of occlusion in deciduous teeth and breastfeeding was assessed in three papers.

The correlation between breastfeeding and the presence of diastemas in deciduous teeth was assessed in three papers [36,38,50]. Only one study [48] evaluated the relationship between maxillofacial growth pattern and breastfeeding in mixed dentition.

### 3.2. Qualitative Synthesis

All the eighteen observational studies scored moderate to high according to the NOS (Table 2).

Limeira et al. [37] observed that the absence or a reduced time of breastfeeding could be a risk factor for posterior crossbite in the mixed dentition. Likewise in the primary dentition, all of the studies apart from Germa et al. 2016 [44] found an increased risk for posterior crossbite and lower bite force if the baby was not breastfed or if it was breastfed for less than 6 months [10,33,38,39,45,49].

As to vertical discrepancy conclusions vary through different studies. Peres et al. [39] and Romero et al. [3] found that anterior open bite was associated with short time or no breastfeeding [33,44]. Moimaz et al. [46] found a greater prevalence of increased overbite in subjects that were breastfed for more than a year. Sum et al. [18], on the contrary, were unable to find any relationship between breastfeeding and vertical discrepancy. 

As to sagittal discrepancy, some studies found an association between longer breastfeeding and reduced overjet [18,39]. However, Moimaz et al. [46] reported increased overjet in subjects with more than a year of breastfeeding. 

Some studies evaluated the association between dental class II and breastfeeding reported that subject breastfed for a longer time were less likely to develop this kind of malocclusion in primary dentition [35] and in mixed dentition [47].

As to occlusion development and breastfeeding, Peres et al. stated that breastfeeding promotes better occlusion [39], Campos et al. [14] reported that children that were not breastfed were more likely to develop malocclusion. On the contrary, Lopes-Freire et al. failed to find any connection between these two variables [15]. Costa et al. [36] stated that subjects that were never or not exclusively breastfed and used a pacifier had worse malocclusion than the ones exclusively breastfed and without not nutritive sucking habits. These authors assumed that the use of pacifier can modify the interaction between occlusal status and breastfeeding.

Three papers [36,38,50] evaluated the association between presence of diastemas in primary dentition and breastfeeding. Chen et al. [38] and Agarwall et al. [38] observed that breastfeeding for up to six months is associated to an absence of maxillary diastemas while Costa et al. associated breastfeeding to diastema and primate spaces [36]. 

Sanchez Molins et al. [48] evaluated the relationship between facial pattern and breastfeeding in mixed dentition and observed more brachyfacial pattern in subjects that have been breastfed.

## 4. Discussion

### 4.1. Breastfeeding as a Prevention Factor for Development of Malocclusions

It could be said that breastfeeding has a preventive effect on the development of malocclusions as it promotes adequate growth and bone and muscle development [39]. Breastfeeding reinforces the physiological nasal breathing of the newborn during and after sucking of breast milk, avoiding oral breathing and thus preventing the development of malocclusions [5,47]. The act of breastfeeding is positively associated with the development of dental arches in the temporal dentition in the anterior transverse and sagittal plane [18,51]. This fact is demonstrated in the study by Sánchez et al. where they compared lateral skull teleradiographs of 197 patients (106 breast-fed and 91 bottle-fed), using the cephalometric values of Ricketts, Steiner and McNamara and concluded that children who received breastfeeding had a correct relationship in the vertical and sagittal plane of the jaw with respect to the maxilla and the cranial base [43,52].

Lescano and Varela [51] examined a sample of 290 5-year-old children, divided into two groups: in group A, children who received breastfeeding during the first months of life, and group B, those who only received artificial lactation. In conclusion, it was obtained that the highest percentage of children with normal occlusion were in group A, who received natural breastfeeding. In addition, it was observed that the cross bite was present in 1.9% of children fed with breast milk, and in a percentage greater than 16.9% in children fed by bottle feeding. Sum et al. conducted a cross-sectional study with a sample of 851 Asian children aged 2 to 5 years in the city of Hong Kong [18]. The parents of the children participating in the study completed a questionnaire to gather information on the use of breastfeeding and non-nutritive sucking habits.

Children breastfed for more than 6 months were less likely to develop a malocclusion in primary dentition, as they concluded that exclusive breastfeeding for more than 6 months is positively associated with the eugnathic development of the maxillomandibular complex both in the transversal and in the sagittal dimension that is children are less likely to develop maxillary hypoplasia and cross-bite and are less likely to develop a distoclusion in primary dentition (that is that the cusp tip of the maxillary primary canine tooth is mesial to the distal surface of the mandibular primary canine) [18]. Gomes et al. [52] carried out a study in Brazil with the objective of measuring and comparing the activity of masseter, temporal and buccal muscles in different forms of infant feeding. To do this, they took a sample of 60 children aged between 2 and 3 months, and distributed them into three groups: (1) Exclusive breastfeeding, (2) Breastfeeding supplemented with artificial feeding by bottle and (3) Breastfeeding supplemented with artificial feeding by cup. All children had a surface electromyography while feeding. With respect to the rate of movement and average contraction of the masseter and temporal muscles, greater activity was observed in the group fed through exclusive breastfeeding compared to the group fed with the bottle. In the case of bucinadores, differences were observed only in the range of contraction of these muscles, being more in the group fed by breastfeeding than in those fed by bottle.

These results suggest that there is similarity in the muscular activity of masseter, temporary and bucinadores in children fed exclusive breastfeeding and even supplemented with cup feeding; Therefore, the latter can be used as an alternative infant feeding method, improving its action on the bottle, due to the hyperactivity of the buccal muscles that could lead to changes in the structural growth and development of the stomatognathic system [53,54].

### 4.2. Duration of Breastfeeding Related to the Appearance of Parafunctional Habits

The scientific literature reports that for children fed by breastfeeding for a period equal to or greater than 6 months, this acts as a prevention factor for the acquisition of harmful oral habits, thanks to the psychological stability obtained by the intimate bond with the calming mother of that instinct of suction [4].

Thomaz et al. (2012), carried out a study in Brazil with a sample of 2060 students aged between 12 and 15, whose results showed the association between a short duration of breastfeeding (less than 6 months) and the development of malocclusions, highlighting Angle class II and a higher incidence of oral breathing in these patients. They concluded that breastfeeding by itself does not cause malocclusions but a synergistic effect can be observed in the presence of parafunctional habits in children who have been breastfed for less than 6 months [47].

In a study conducted by Lopes et al. (2015), the influence of breastfeeding on the development of non-nutritive sucking habits such as digital sucking and prolonged use of the pacifier was studied in a population of 275 children aged between 3 and 6, of the which 28 had received exclusive breastfeeding and 247 mixed breastfeeding. The presence of parafunctional habits was observed in 224 children (81.5%). Among the results obtained, it should be noted that children who received exclusive breastfeeding did not have parafunctional habits [15]. Leite et al. (2007), conducted a study with the objective of relating the type of breastfeeding received with the development of non-nutritive sucking habits and malocclusions [53]. The sample consisted of 342 children aged between 3 and 5 years of age and observed that non-nutritive sucking habits had a high prevalence of 70 to 77.4% of the population studied. The malocclusions were present in 87% of the patients.

An proportion of 84.2% of the children reported having received breastfeeding and of these 79.9% presented some evidence of malocclusion at the time of the clinical examination. These authors conclude that there is a significant relationship between the period of breastfeeding, the continuation with artificial lactation and the appearance of non-nutritive sucking habits in children, and that this variable is strongly associated with the development of malocclusions. Of the 70 children who were fed by breastfeeding for a period equal to or greater than 19 months, 65.7% (n = 46) had no deleterious oral habits [53]. Morales et al. [54], carried out a study in Caracas with the objective of evaluating the association between a short breastfeeding period of less than 6 months and the development of parafunctional habits analyzing 195 medical records of patients between the ages of 3 and 16 years old. The type of breastfeeding received, breastfeeding time, presence of harmful oral habits such as digital sucking, pacifier use, lingual interposition, bruxism, atypical swallowing and dyslalias and presence of malocclusions were investigated [55,56]. The authors observed that there was a direct relationship between breastfeeding time less than 6 months and development of parafunctional habits, the risk being greater for those children who did not receive breastfeeding [4].

### 4.3. Bottle Feeding as an Etiological Factor and Promoter of Malocclusions and Harmful Oral Habits

Artificial breastfeeding using a bottle, used as an alternative method to breastfeed, predisposes according to the scientific literature to the development of malocclusion.

Mendoza et al. 2008 [57], carried out a study with a sample of 500 Bolivian children aged between 3 and 7 years. The authors observed that breastfeeding during the first six months of life is represented as a prevention factor for the development of malocclusions. However, artificial feeding is represented as a risk factor for their development. It was observed that bottle-fed children had a 64% prevalence of non-nutritive sucking habits, the most frequent being digital suction with 53%, followed by pacifier suction with 28% and other habits such as lingual interposition and lipstick in 19%. They concluded that artificial feeding associated with non-nutritive sucking habits are the main risk factors that lead to the likelihood of developing malocclusions.

Moimaz et al. (2008), developed a study with a sample of 100 children under one year of age whose purpose was to evaluate the relationship on the type of feeding of infants and the development of non-nutritive sucking habits [46]. The results showed that 75% of the children were being fed through breastfeeding. The habits of digital sucking and sucking of the pacifier were obtained in 55% of the children, these habits being present in 74% of the children who were fed by bottle. These results suggest the hypothesis that artificial feeding can be considered a risk factor in the occurrence of non-nutritive sucking habits in children.

Chen et al. (2015), conducted a study with a sample of 734 children in Beijing where they observed how artificial breastfeeding time influences the development of malocclusions [38]. Children who were fed by the bottle for a period of time greater than 18 months have a risk greater than 1.6%, 1.16% and 1.43% of having, respectively, posterior cross bite, maxillary compression and canine Class II than children who received said feeding until 18 months.

In spite of being an efficient alternative of feeding in the infant, artificial lactation can give rise to an insufficient mandibular development due to a minimum functional requirement at the time of the feeding, since this last one is realized from a rigid material, inducing patterns of low muscle activity, causing transverse growth of the palate and inadequate dental alignment, situations that demonstrate a strong relationship with the presence of dental and skeletal malocclusions.

## 5. Conclusions

It appears a rather common finding in the literature that breastfeeding for 6 months or more reduces the risk for posterior crossbite and class II malocclusion in primary and mixed dentition. However, no clear evidence exists of breastfeeding being protective against other types of malocclusion (e.g., vertical discrepancy like open bite or deep bite). Prospective longitudinal studies with data on duration and on other characteristics of breastfeeding (e.g., exclusive or mixed breastfeeding, association with not nutritive sucking habits and so forth), and subsequent evaluation of the occlusal status during primary dentition, mixed dentition and permanent dentition would greatly help in reducing biases and confusing factors such as non-nutritional sucking habits.

## Figures and Tables

**Table 1:** Included studies characteristics.

Author	Publication Date	Design	Journal	Sample Size	Subjects’Age at the Diagnosis of Malocclusion	Orthodontic Diagnosis	Malocclusion Types Considered	Assessment Method	Statistical Analysis
Campos MPMS et al. [14]	2018	Cross-sectional study	*Braz Oral Res.*	290	6 years	Clinical examination. Anterior open bite was diagnosed based on the criteria given by Foster and Hamilton [43].	Dental and skeletal malocclusions were evaluated.	The Z-score development index (ratio between height and age) was employed to express nutritional status at birth and at 6 years of age (WHO reference standard).	Multivariate logistic regression; odds ratio (OR), chi-square test.
Costa CTD. et al. [36]		Cross-sectional study	*Braz Oral Res*.	489	2–5 years	WHO index	Anterior crossbite, open bite, median line deviation, crowding or spacing	Validated questionnaire.	Bivariate and multivariable logistic regression, odds ratio (OR)
Germa et al. [44]	2016	Cohort study	*Angle Orthod.*	422	3 years	Clinical examination. Direct inspection for transverse and vertical relation.	Anterior open bite, posterior crossbite.	Self-administered questionnaires	Multiple logistic regressions.
Lopes-Freire GM et al. [15]	2015	Cross-sectional observational survey	*Prog Orthod.*	275	3–6 years	Clinical examination. Direct inspection for transverse and vertical relation. Angle class/primary teeth canine relationship for interarch sagittal malocclusion.	Posterior crossbite. anterior open bite, and overbite, sagittal occlusal relationship (angle class, primary canine relationship, overjet)	Validated questionnaire.	Chi-square, Fisher’s exact tests, odds ratio (OR)
Peres KG. et al. [39]	2015	Cohort study	*Pediatrics*	1303	5 years	Clinical examination in a single home visit. WHO classification	Openbite; crossbite; overjet.	Questionnaire recorded at 3, 12, and 24 months.	Poisson regression analyses
Chen X et al. [38]	2015	Cross- sectional study	*BMC Pediatr.*	734	3–6 years	Does not specify	Deep bite, open bite, anterior/posterior crossbite, sagittal relationship between primary canines and between second primary molars, presence or absence of crowding or spacing.	Questionnaire repeated every six months for the first three years of life and the non-nutritive sucking habits.	Univariate analysis and multiple logistic regressions
Sum FH et al. [18]	2015	Cross-sectional study	*BMC Oral Health*	851	2–5 years	Own criteria (Kappa 0.70–1.00)	Incisal relationship; sagittal relationship between primary canines and between second primary molars, overjet; anterior/posterior crossbite; anterior openbite; overbite; intercanine width; intermolar width; posterior crossbite	Self-administered questionnaires on feeding habits and history of non-nutritive sucking habits	Multinomial logistic regression models; Multi-way ANOVA.
Agarwal SS et al. [45]	2014	Cross-sectional retrospective study	*Prog Orthod.*	415	4–6 years	Self-defined criteria statistically validated (kappa = 0.758)	Anterior open bite, posterior crossbite upper and lower inter-molar distance (IMD) and inter-canine distance (ICD)	One-time administered validated questionnaire	Chi-square test and Odds ratio to assess the strength of correlations in object. Multivariate logistic regression to determine independent predictors of posterior crossbite and upper and lower IMD and ICD
Limeira et al. [37]	2014	Cross-sectional retrospective study	*J Dent Child.*	714	6 to 9 years old	The clinical examination was performed with the subject seated in a chair under natural light, using disposable tongue blades and gloves. The occlusal relationships were evaluated in centric occlusion.	Posterior cross-bite	A validated questionnaire was completed by guardians concerning the length of time they were exclusively breast-fed and the breast-feeding duration.	Chi-square test.
Moimaz SA et al. [46]	2014	Prospective cohort study	*BMC Oral Health*	80	30th months of age	Not reported	Overjet, Posterior crossbite	Self-administered questionnaires at one year, one and a half years and two years of age	Chi-squared and Fisher’s exact tests
Caramez da Silva et al. [35]	2012	Cross-sectional study	*Breastfeed Med*.	153	3 to 5 years old	The sagittal relation between the upper and lower jaw was evaluated through direct clinical examination Distoclusion was diagnosed according to Foster and Hamilton’s criteria [43] (the cusp of the primary upper canine occluded anterior to the distal aspect of the primary lower canine.	Primary teeth sagittal relationship	Trained research assistants gathered data on dietary and not-nutritive sucking habits at 7, 30, 60, 120, and 180 days through a telephonic or in person interview (if the first option was not viable)	Poisson’s regression analysis.
Thomaz et al. [47]	2012	Cross-sectional study	*Int J Pediatr otorhinolaryngol*.	2060	12–15 years old	Malocclusion and facial characteristics were evaluated as defined by Angle	Dental class as described by Angle	Validated questionnaire.	Odds ratio (OR) in multinomial logistic regression analysis
Jabbar NS et al. [2]	2011	Epidemiological study, cross-sectional study	*Braz Oral Res*.	911	3–6 years	Self-defined criteria statistically validated (Kappa: 0.9 to 1.0)	Overjet (normally, increased, anterior crossbite). Primary canine relationships (Class 1,2,3)	One-time validated questionnaire.	multiple binary logistic regression (α = 0.05)
Romero CC et al. [3]	2011	Cross-sectional study	*J Appl Oral Sci*	1377	3–6 years	Clinical examinations were performed by visual inspection.	Overbite alterations: anterior open bite (negative overbite) and anterior deep bite (increased overbite)	Validated questionnaire.	Spearman’s correlation test., chi-square tests with odds ratio (OR), binary logistic regression
Sanchez Molins et al. [48]	2010	Observational, analytical and retrospective study	*Eur J Paediatr Dent*	197	6–11years	Cephalometric measurements according to Ricketts, Steiner and McNamara.	Dental, skeletal and aesthetics variables based on Ricketts, Steiner and McNamara values.	Validated questionnaire.	t-test and ANOVA test, chi-square test
Castelo PM et al. [49]	2010	Cross-sectional study	*J Appl Oral Sci*.	67	3.5 to 7 years	Direct clinical examination of allowed to gather the following information: anamneses, height and weight, posterior crossbite, distoclusion was diagnosed according to Foster and Hamilton’s criteria [43] (the cusp of the primary upper canine occluded anterior to the distal aspect of the primary lower canine).	Cross-bite, maximal bite force.	Direct clinical examination and interview of the guardians about history of breastfeeding, presence and duration of sucking habits.	t-test, Pearson’s correlation test multiple logistic regression univariate regression
Peres KG et al. [33]	2007	Cross-sectional study	*Rev Saude Publica.*	359	Not reported	Direct clinical examination	Anterior open bite and posterior cross bite	Repeated interview of the guardians about breastfeeding and non-nutritive sucking habits were performed at birth, after 3, 6 and 12 months, and at six years of age	Chi-square test, Poisson regression test
Viggiano D et al. [10]	2004	Retrospective study	*Arch Dis. Child*.	1130	3–5 years	Direct clinical examination by a pediatric dentist	Altered sagittal relationship; anterior open bite; posterior cross-bite	Structured ques-tionnaire	Logistic regression, odds ratio

Significance threshold was set for all studies at *p* < 0.05.

**Table 2:** Quality scores of included studies according to Newcastle-Ottawa Quality Assessment Scale (NOS) for cohort studies.

Author (Year)	Selection (****)				Comparability (**)		Outcome (***)			Total Score
	1	2	3	4	5a	5b	6	7	8	
Campos et al.(2018) [14]	*	*					*	*		4
Costa et al. (2018) [36]				*			*	*		3
Germa et al.(2016) [44]	*	*					*	*	*	5
Lopes-Freire et al. (2015) [15]	*	*	*				*	*		5
Peres et al. (2015) [39]	*	*	*				*	*	*	6
Chen et al. (2015) [38]	*	*					*	*		4
Sum et al. (2015) [18]	*	*					*	*		4
Agarwal et al. (2014) [45]	*	*					*	*		4
Limeira et al. (2014) [37]	*	*		*			*	*	*	6
Moimaz et al. (2014) [46]	*	*	*				*	*	*	6
Caramez da Silva et al. (2012) [35]	*	*					*	*	*	5
Thomaz et al. (2012) [47]	*	*					*	*	*	5
Jabbar et al. (2011) [2]	*	*					*	*	*	5
Romero et al.(2011) [3]	*	*					*	*		4
Sanchez-Molins et al. (2010) [48]	*	*					*	*		4
Castelo et al. (2010) [49]	*	*	*				*	*		5
Peres et al. (2007) [33]	*	*	*				*	*	*	6
Viggiano et al. (2004) [10]	*	*	*				*	*		5

Description of NOS points: (1) Representativeness of the exposed cohort. (2) Selection of the non-exposed cohort. (3) Ascertainment of exposure. (4) Demonstration that outcome of interest was not present at start of study. (5) Comparability of cohorts on the basis of the design or analysis, (5a) for one factor and (5b) for additional factor. (6) Assessment of outcome. (7) Duration of follow-up period. (8) Adequacy of follow-up.
